# Optimizing endoscopic detection of early gastric cancer: stratification and preventive strategies for Peri-ESD diagnostic oversights

**DOI:** 10.3389/fonc.2026.1745307

**Published:** 2026-02-10

**Authors:** Lei Zhang, Nana An, Xiuli Zheng, Wenqian Ma, Juntao Lu, Limian Er

**Affiliations:** 1 Department of Endoscopy, the Fourth Hospital of Hebei Medical University, Shijiazhuang, Hebei, China; 2 Laboratory of Pathology, Hebei Cancer Institute, the Fourth Hospital of Hebei Medical University, Shijiazhuang, Hebei, China

**Keywords:** AG, atrophic gastritis, EGC, early gastric cancer, IM, intestinal metaplasia, NF-NBI, near-focus narrow-band imaging, Peri-ESD MEGCs, peri-endoscopic submucosal dissection missed early gastric cancers

## Abstract

**Background:**

Peri-endoscopic submucosal dissection missed early gastric cancers (peri-ESD MEGCs), defined as ESD-indicated lesions overlooked during pre-ESD diagnostic workup or post-ESD surveillance, may arise from deficiencies at any procedural phase (preparation, observation, diagnosis, or sampling). To address this, we developed a temporal-procedural bidirectional assessment protocol specifically targeting peri-ESD MEGCs, aiming to optimize endoscopic quality and prevent diagnostic omissions.

**Methods:**

In this retrospective cohort study, 1,011 EGC lesions treated with endoscopic submucosal dissection (ESD) between 2017 and 2024 were analyzed. The primary analysis defined the MEGC time window as 24 months. Peri-ESD MEGCs were stratified into two temporal phases, pre-ESD examination vs. post-ESD surveillance, and four etiological categories, inadequate preparation, inadequate observation, diagnosis error, and sampling error, for each endoscopic cause of MEGC. To assess the robustness of our findings, a sensitivity analysis was performed by redefining the MEGC time window as 12 months.

**Results:**

Among 94 peri-ESD MEGCs, pre-ESD MEGCs (n=52) predominantly demonstrated inadequate observation (51.9%), which was associated with greater curvature location (OR: 5.45; 95% CI: 1.76–16.91), nonuse of near-focus narrow-band imaging (NF-NBI, OR: 16.78; 95% CI: 5.50–51.26), and severe intestinal metaplasia (OR: 3.84; 95% CI: 1.09–13.52). Post-ESD MEGCs (n=42) predominantly demonstrated a diagnosis error (52.4%), correlated with trainees (OR: 3.53; 95% CI: 1.06–11.68), small lesions (<15 mm, OR: 3.83; 95% CI: 1.10–13.36), nonuse of NF-NBI (OR: 17.44; 95% CI: 4.81–63.17), and severe atrophic gastritis (OR: 7.78; 95% CI: 1.91–31.20). The sensitivity analysis using a 12-month MEGC time window yielded results consistent with the primary analysis, demonstrating the robustness of the identified risk factors for peri-ESD missed lesions.

**Conclusions:**

Optimizing peri-ESD gastroscopic observation (via NF-NBI) and post-ESD diagnostic accuracy (through operator training) could significantly reduce peri-ESD MEGCs, particularly those reflecting characteristics of ESD-eligible lesions.

## 1 Introduction

Endoscopic submucosal dissection (ESD) remains the gold standard for lymph node-negative early gastric cancer (EGC) management (1), achieving favorable outcomes when applied to strictly selected cases per guidelines (2). However, the reported 0.9%-19.0% incidence of peri-ESD missed early gastric cancers (peri-ESD MEGCs, defined as ESD-indicated lesions overlooked during pre-ESD examination or post-ESD surveillance) significantly elevates disease progression risk (3–7).This necessitates enhanced endoscopic detection precision to avert tumor staging migration and unnecessary radical resection.

Current classification systems for peri-ESD MEGCs remain both inconsistent and oversimplified (5), failing to capture their occurrence across various phases (pre-ESD to post-ESD) and procedural steps (preparation, observation, diagnosis, sampling). To address this complexity, our team has therefore implemented a temporal–procedural bidirectional assessment protocol specifically tailored to evaluate peri-ESD MEGCs.

The primary objective was to integrate clinical parameters through this protocol to optimize endoscopic examination quality and precisely prevent diagnostic omissions in the peri-ESD workflow.

## 2 Methods

### 2.1 Patients

**Figure 1:**
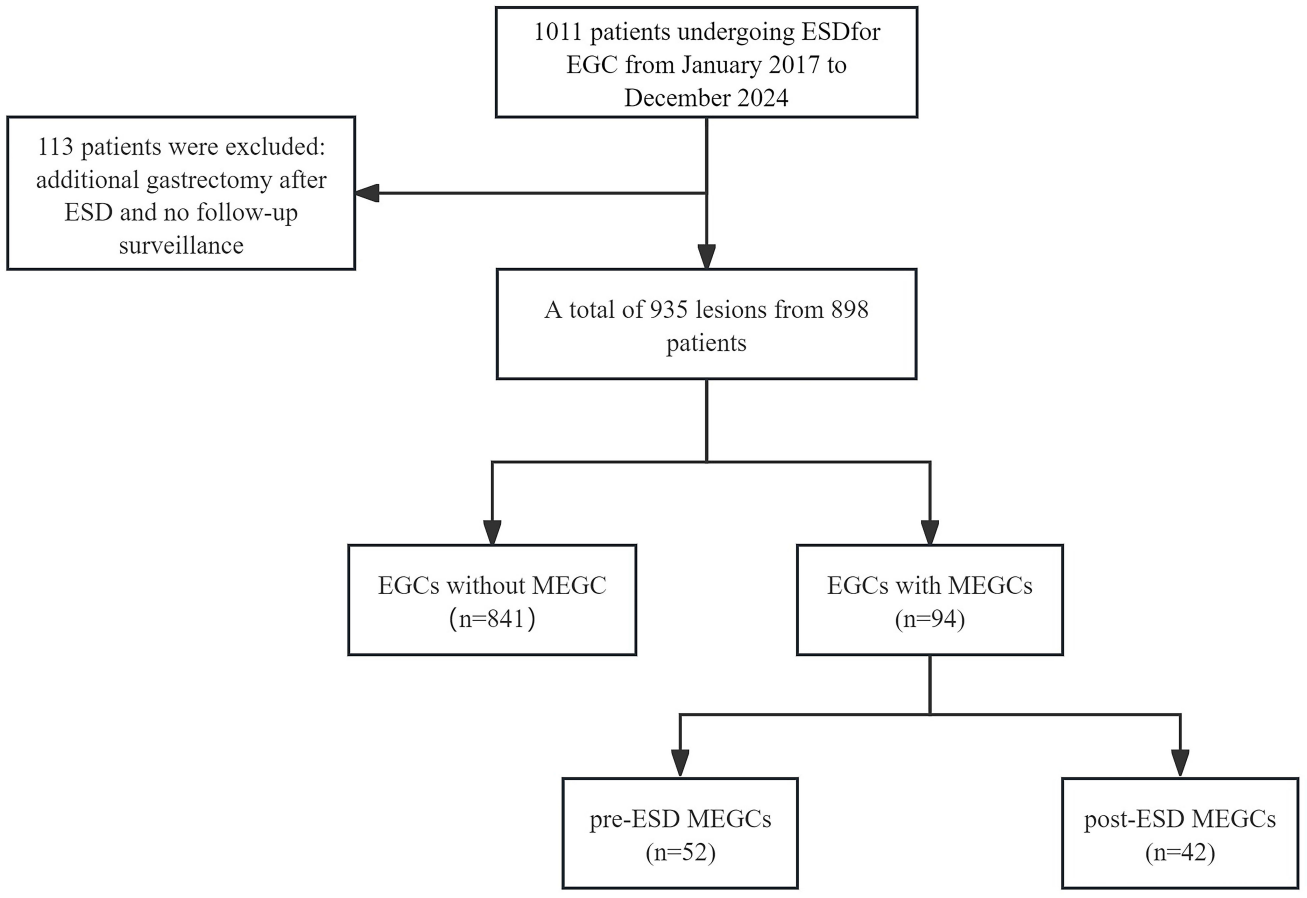
Patient flowchart. ESD, endoscopic submucosal dissection; EGC, early gastric cancer; MEGCs, missed early gastric cancers.

In this retrospective analysis, 1011 patients who underwent ESD for primary EGC at Hebei Medical University Fourth Hospital from January 2017 to December 2024 were included initially. The exclusion criteria included patients requiring post-ESD gastrectomy (n=87) and those lost to follow-up (n=26), yielding a final cohort of 898 participants (**Figure 1**). The studies involving humans were approved by the Ethics Committee of the Fourth Hospital of Hebei Medical University (2024KY230), the studies were conducted in accordance with the local legislation and institutional requirements. The participants provided their written informed consent to participate in this study. The original contributions presented in the study are included in the article/**Supplementary Material**. Further inquiries can be directed to the corresponding author.

To facilitate examination of the mucosal surface, patients were instructed to ingest a solution containing mucolytic and defoaming agents orally prior to the procedure. The formulation used consisted of 100 mL of water containing 20,000 units of streptokinase (Tide Pharmaceutical, Beijing, China) and 5 g of dimethicone powder (Honghe Pharmaceutical, Sichuan, China).

Endoscopy was conducted using endoscopes (GIF-H260, GIF-HQ290; Olympus Medical, Tokyo, Japan) with propofol anesthesia administered intravenously. For patients unable to receive intravenous anesthesia, dyclonine hydrochloride mucilage was administered for pharyngeal anesthesia. During endoscopic procedures, food residue and mucus in the stomach were meticulously removed to ensure clear observation of the mucosal surface.

An expert consensus opinion on standardized endoscopic resection of early gastric cancer was reached in 2018 (8). Absolute indications (1): differentiated intramucosal carcinoma without ulcers (cT1a); (2) differentiated intramucosal carcinoma (cT1a) with lesion sizes ≤3 cm and ulcers; (3) high-grade gastric intraepithelial neoplasia (HGIN). Expanded indication: undifferentiated intramucosal carcinoma (cT1a) with a lesion size ≤2 cm and no ulceration. Post-ESD surveillance included assessments at 3 months, 6 months, 12 months, and then annually thereafter.

### 2.2 Endoscopic Examination Protocol

1. Pre-procedural Preparation: The standard pre-examination oral medication and sedation protocol for patients.
2. Systematic Examination Procedure: A defined systematic observation sequence from the esophagus, gastric body, to the duodenum, with particular emphasis on the examination techniques, irrigation requirements, and minimum observation times for high-risk areas such as the greater curvature location.
3. Image Acquisition Standards: Clear specifications regarding the number of images and anatomical sites.
4. Biopsy Protocol: Guidance that upon detection of suspicious lesions, targeted biopsies may be obtained with the assistance of near-focus narrow-band imaging (NF-NBI).

### 2.3 NF-NBI activation criteria

1. WLI confirmation of mucosal abnormalities required prior to mode switching;
2. Fixed 45× magnification applied for lesion characterization;
3. Biopsy mandated when microstructural irregularities are identified, pending pathological confirmation.

### 2.4 Image screening process

Two endoscopists (Mingli Wu and Zhibin Xu, board-certified fellows of the Anti-Cancer Association of Hebei Province) who were blinded to the clinicopathologic information independently reviewed the endoscopic images taken according to the observation system. They carried out a retrospective analysis of the images of 898 patients who underwent ESD for early gastric cancer and were screened for missed diagnoses before ESD, namely, during preoperative examination, and after ESD, namely, during postoperative review. If the diagnoses were not identical, a consensus was reached after the endoscopic images were reviewed again.

### 2.5 Definition

1. Temporal–procedural bidirectional assessment protocol.

**Figure 2:**
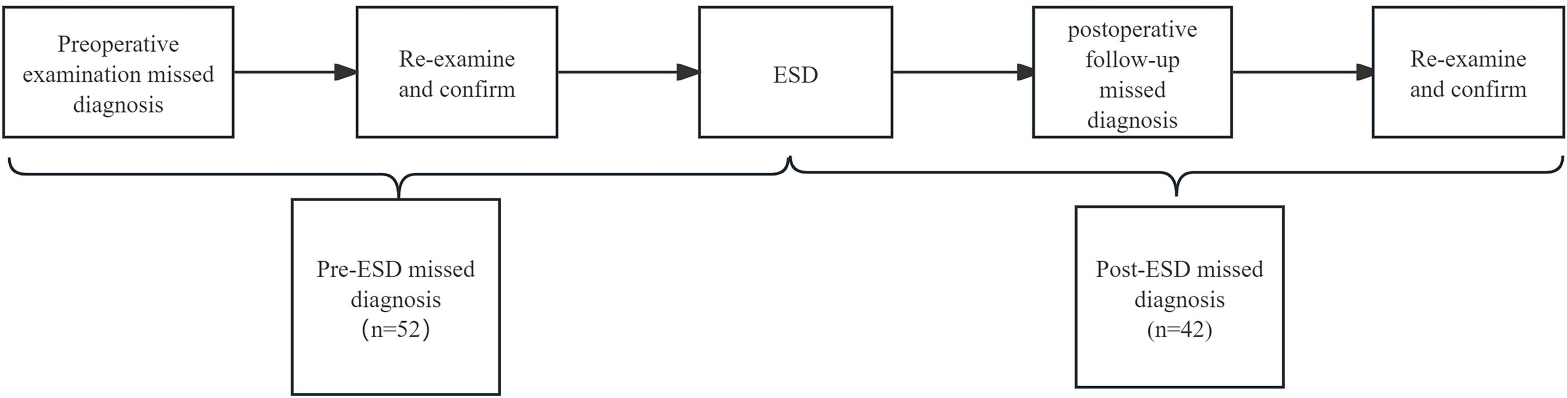
Process of endoscopic MEGCs. MEGCs, missed early gastric cancers; ESD, endoscopic submucosal dissection.

Temporal axis: Stratifies missed diagnoses based on detection timeline relative to the peri-ESD period (pre- vs post-ESD phases). Procedural axis: Identifies root causes through systematic analysis of endoscopic workflow breakdowns (preparation→ observation→ diagnosis→ sampling). (**Figure 2**).

2. Temporal axis:

The study cohort was stratified into three diagnostic categories based on temporal identification patterns: EGCs with MEGCs and EGCs without MEGCs.

2.1 EGCs with MEGCs.

1. MEGCs detected during pre-ESD examinations (pre-ESD MEGCs): Defined by retrospective analysis of preoperative endoscopic imaging demonstrating previously undetected lesions subsequently confirmed through histopathological review during secondary evaluations.
2. MEGCs detected during post-ESD surveillance (post-ESD MEGCs): Characterized by interval development of metachronous lesions with retrospective analysis of postoperative endoscopic imaging that revealed surveillance deficiencies following ESD and followed by histopathological confirmation obtained during secondary evaluations, regardless of prior diagnostic status.

2.2 EGCs without MEGCs.

EGCs without MEGCs: Comprised cases with complete endoscopic–pathological correlation at initial presentation, exhibiting no evidence of diagnostic oversight upon blinded expert review of baseline endoscopic records.

3. Procedural axis:

1. Inadequate observation: The absence of lesions or a lack of focused images that resulted in incomplete lesion assessment; This could occur because the lesion was missed due to its anatomical location or an oversight by the original endoscopist during the examination, as evidenced by the absence of images of the specific area or the presence of only non-diagnostic, unfocused images.
2. Inadequate preparation: A significant amount of food residue or mucus that was unable to be removed and prevented adequate observation;
3. Diagnosis error: Morphologically evident lesions that lacked formal pathological evaluation despite retrospective recognition; This could result from the original endoscopist’s inexperience in identifying the lesion’s suspicious morphology or from a misinterpretation (e.g., classifying a lesion as benign based on NF-NBI features despite its malignant nature).
4. Sampling error: Initial histopathological misinterpretation of malignant lesions that required confirmatory biopsy verification.

Comprehensive endoscopic records spanning 24 months before and after ESD were subjected to systematic analysis.

4. Clinical data.

The analysis factors included patient demographics (age, gender), operator expertise, lesion characteristics (site, size, classification), categorization of MEGC, assessment of mucosal visibility, quantity of image acquisition, NF-NBI observations, degree of atrophic gastritis (none, C-1, C-2, C-3, O-1 and above), intestinal metaplasia (none, mild or severe), and degree of gastric mucosa inflammation (none, mild or severe). Postoperative pathology findings were also included in the analysis.

Detailed Explanation of Relevant Factors: Endoscopists were categorized into experts and trainees. The experts were defined as endoscopists who performed more than 1,000 endoscopic examinations per year on average during the study period, as well as those who independently performed ESD, whereas the trainees were defined as endoscopists who completed an average of fewer than 1,000 endoscopies per year during the study period and were unable to complete ESD.

Mucosal visibility was stratified into three distinct grades (high, medium, low) on the basis of a validated scoring system (1–4 points) adapted from Kuo et al. (9). High-grade visibility denotes mucosal surfaces devoid of adherent mucus. Medium-grade visibility encompasses either minimal mucus deposition without visual obstruction or substantial mucus requiring limited water irrigation (<50 mL) for clearance. Low-grade visibility indicates dense mucus coatings necessitating extensive irrigation (>100 mL) or demonstrating complete irremovability.

Endoscopic image documentation was quantitatively stratified into two cohorts: ≥40 images (comprehensive capture) and <40 images (limited capture). Endoscopic atrophy was evaluated on the basis of the Kimura–Takemoto classification (10): mild (C0 - C1), moderate (C2 - C3), and severe (O1 - O3). Similarly, the severity of intestinal metaplasia was classified as mild (no metaplasia or mild intestinal metaplasia), moderate (moderate intestinal metaplasia), or severe (severe intestinal metaplasia).

Endoscopic manifestations of *Helicobacter pylori*-associated gastritis, including mucosal edema and erythema, were diagnostically confirmed through standardized imaging protocols (11, 12). Zhao et al.’s (13) operationally defined “ current active *H. pylori* infection” as the endoscopic presence of ≥1 diagnostic criterion: nodularity, diffuse erythema, or mucosal edema. Given the absence of direct *H. pylori* serological data, our retrospective cohort analysis of endoscopic archives enabled the identification of severe gastric mucosal inflammatory patterns (triad: nodularity, diffuse erythema, edema), which served as surrogate markers for current *H. pylori* infection status. These parameters were incorporated into our analytical framework.

### 2.6 Statistical analysis

The statistical analysis was conducted using the SPSS 21.0 software (IBM SPSS Statistics, IBM Corporation, Armonk, NY) for MS Windows. Categorical variables are presented as absolute numbers or percentages; continuous data are expressed as means with corresponding standard deviations; univariate analysis involved the use of the Pearson χ^2^ test or Fisher exact test for categorical data; multivariate analysis was carried out through logistic regression; and differences in the data with P values < 0.05 were considered statistically significant. A sensitivity analysis was performed by redefining the MEGC time window as 12 months (instead of the primary 24-month definition) to test the robustness of our findings.

## 3 Results

**Table 1** summarizes the clinicopathologic characteristics of the study cohort. In this study, a total of 935 lesions from 898 patients were included. Among the participants, 714 were male and 184 were female. In this cohort, 94 lesions from 86 patients were missed, including 52 pre-ESD lesions and 42 post-ESD lesions from 37 patients. Moreover, lesions from 3 patients were missed both pre-ESD and post-ESD, resulting in an overall miss rate of 10.1%.

**Table 1:** Clinicopathologic characteristics of missed early gastric cancers and EGCs without MEGCs.

Clinical parameters	EGCs without MEGCs (n=841)	Pre-ESD MEGCs (n=52)	Post-ESD MEGCs (n=42)
Sex			
Male	637 (78.4%)	46 (88.5)	34 (80.9%)
Female	175 (21.6%)	6 (11.5%)	3 (19.1%)
Age			
<65years old	455 (44.0%)	30 (57.7%)	14 (33.3%)
≥65years old	357 (56.0%)	22 (42.3%)	23 (66.7%)
Endoscopist			
expert	326 (38.8%)	21 (40.4%)	20 (47.6%)
trainee	515 (61.2%)	31 (59.6%)	22 (52.4%)
Lesion size			
<1.5cm	373 (44.4%)	35 (67.3%)	26 (61.9%)
≥1.5cm	468 (55.6%)	17 (32.7%)	16 (38.1%)
Lesion classification			
I	13 (1.5%)	2 (3.8%)	0 (0.0%)
IIa	93 (11.1%)	8 (15.4%)	7 (16.7%)
IIb	49 (5.8%)	10 (19.2%)	6 (14.3%)
IIc	521 (62.0%)	25 (48.1%)	26 (61.9%)
IIa+IIc	165 (19.6%)	7 (13.5%)	3 (7.1%)
Site 1			
Upper third	576 (68.5%)	32 (61.5%)	28 (66.7%)
Middle third	143 (17.0%)	9 (17.3%)	10 (23.8%)
Lower third	122 (14.5%)	11 (21.2%)	4 (9.5%)
Site 2			
Lesser curvature	408 (48.5%)	22 (42.3%)	16 (38.1%)
Greater curvature	66 (7.8%)	14 (26.9%)	8 (19.0%)
Anterior wall	98 (11.7%)	10 (19.3%)	5 (11.9%)
Posterior wall	269 (32.0%)	6 (11.5%)	13 (31.0%)
Mucosal visibility			
Low	10 (1.2%)	1 (1.9%)	1 (2.3%)
Medium	125 (14.9%)	17 (32.7%)	13 (31.0%)
High	706 (83.9%)	34 (65.4%)	28 (66.7%)
Quantity of image acquisition			
<40photos	365 (43.4%)	26 (50.0%)	22 (52.4%)
≥40photos	476 (56.6%)	26 (50.0%)	20 (47.6%)
NF-NBI			
nonuse of NF-NBI	162 (19.3%)	38 (73.1%)	31 (73.8%)
NF-NBI	679 (80.7%)	14 (26.9%)	11 (26.2%)
Atrophic gastritis			
Mild	538 (64.0%)	28 (53.8%)	9 (21.4%)
Moderate	185 (22.0%)	11 (21.2%)	12 (28.6%)
Severe	118 (14.0%)	13 (25.0%)	21 (50.0%)
Intestinal metaplasia			
Mild	683 (80.9%)	32 (61.5%)	22 (52.4%)
Moderate	74 (9.2%)	8 (15.4%)	8 (19.0%)
Severe	84 (10.0%)	12 (23.1%)	12 (28.6%)
Gastric mucosal inflammation			
Mild	557 (66.2%)	38 (73.1%)	19 (45.2%)
Severe	284 (33.8%)	14 (26.9%)	23 (54.8%)
Histologic type			
Differentiated	650 (77.3%)	45 (86.5%)	37 (88.1%)
Undifferentiated	191 (22.7%)	7 (13.5%)	5 (11.9%)

### 3.1 Incidence and endoscopic causes of MEGCs

#### 3.1.1 Pre-ESD missed lesions

**Figure 3:**
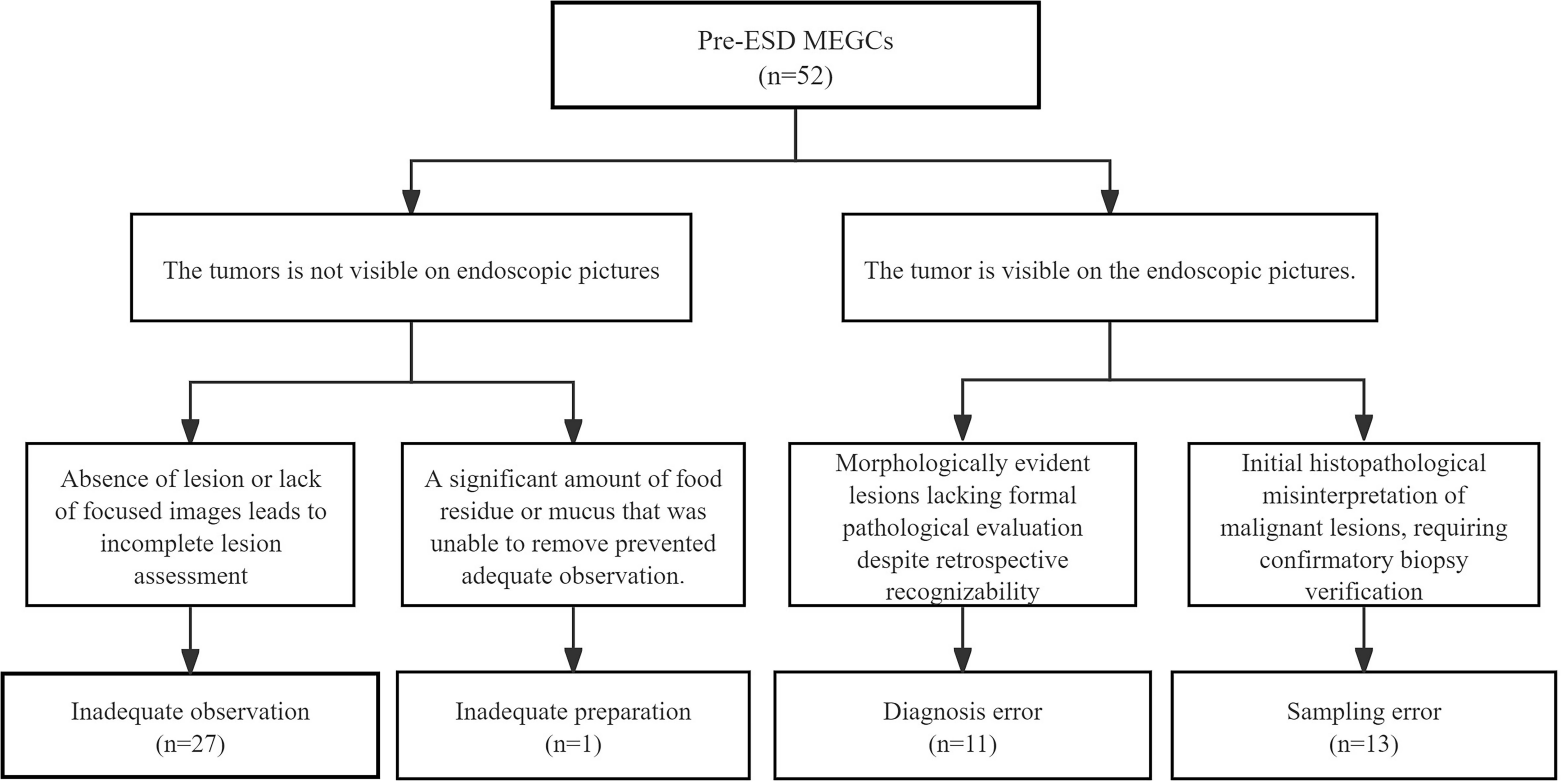
Classification of MEGCs pre-ESD. MEGC, missed early gastric cancer. ESD, endoscopic submucosal dissection.

Using the primary 24-month definition, we identified 52 pre-ESD MEGCs. Of these, 27 (51.9%), 1 (1.9%), 11 (21.2%), and 13 (25.0%) were attributed to inadequate observation, inadequate preparation, diagnosis error, and sampling error, respectively (**Figure 3**).

A sensitivity analysis was performed by redefining the MEGC time window as 12 months, which identified 35 pre-ESD MEGCs. The distribution of causes under this more stringent definition remained consistent: 18 (51.4%), 1 (2.9%), 9 (25.7%), and 7 (20.0%) were due to inadequate observation, inadequate preparation, diagnosis error, and sampling error, respectively. This demonstrates the stability of our endoscopic cause classification.

#### 3.1.2 Post-ESD missed lesions

**Figure 4:**
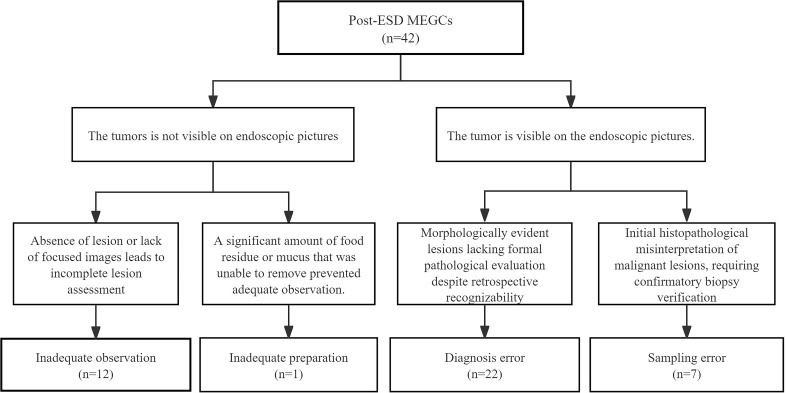
Classification of MEGCs post-ESD. MEGC, missed early gastric cancer. ESD, endoscopic submucosal dissection.

Among the 42 MEGCs detected post-ESD within the 24-month window, the causes were distributed as follows: 12 (28.6%) from inadequate observation, 1 (2.4%) from inadequate preparation, 22 (52.4%) from diagnosis error, and 7 (16.6%) from sampling error (**Figure 4**).

The sensitivity analysis with a 12-month window identified 23 post-ESD MEGCs. The pattern of primary causes was similarly reaffirmed, with 8 (34.8%) attributed to inadequate observation, 0 (0.0%) to inadequate preparation, 11 (47.8%) to diagnosis error, and 4 (17.4%) to sampling error.

### 3.2 Risk factors for MEGCs

#### 3.2.1 Risk factors for pre-ESD inadequate observation

Multivariate analysis under the primary 24-month definition identified significant associations between pre-ESD inadequate observation and lesion location on the greater curvature (OR: 5.45; 95% CI: 1.76–16.91), nonuse of NF-NBI (OR: 16.78; 95% CI: 5.50–51.26), and the presence of severe intestinal metaplasia (OR: 3.84; 95% CI: 1.09–13.52) when compared to the control group of EGCs without MEGCs (**Table 2**).

**Table 2:** Logistic regression analysis of risk factors associated with inadequate observation pre-ESD.

Clinical parameters	EGCs without MEGCs (n=841)	Inadequate observation (n=27)	Chi-square	Multivariate analysis	
			P	Odds ratio (95% confidence interval)	P
Sex					
Male	637 (78.4%)	23 (85.2%)	0.483		
Female	175 (21.6%)	4 (14.8%)			
Age					
<65years old	455 (44.0%)	17 (63.0%)	0.557		
≥65years old	357 (56.0%)	10 (37.0%)			
Endoscopist					
trainee	326 (38.8%)	15 (55.6%)	0.108		
expert	515 (61.2%)	12 (44.4%)			
Lesion size					
<1.5cm	373 (44.4%)	19 (70.3%)	0.010	2.45 (0.95-6.34)	0.064
≥1.5cm	468 (55.6%)	8 (29.7%)			
Lesion classification					
I	13 (1.5%)	0 (0.0%)	0.003		
IIa	93 (11.1%)	1 (3.7%)			
IIb	49 (5.8%)	8 (29.6%)			
IIc	521 (62.0%)	14 (51.9%)			
IIa+IIc	165 (19.6%)	4 (14.8%)			
Site 1					
Upper third	576 (68.5%)	13 (48.2%)	0.062		
Middle third	143 (17.0%)	7 (25.9%)			
Lower third	122 (14.5%)	7 (25.9%)			
Site 2					
Lesser curvature	408 (48.5%)	10 (37.1%)	0.000		
Greater curvature	66 (7.8%)	9 (33.3%)		5.45 (1.76-16.91)	0.003
Anterior wall	98 (11.7%)	5 (18.5%)		2.37 (0.65-8.61)	0.189
Posterior wall	269 (32.0%)	3 (11.1%)		0.51 (0.13-2.02)	0.337
Mucosal visibility					
Low	10 (1.2%)	1 (3.7%)	0.011		
Medium	125 (14.9%)	9 (33.3%)		1.32 (0.11-15.72)	0.826
High	706 (83.9%)	17 (63.0%)		0.77 (0.07-78.62)	0.772
Quantity of image acquisition					
<40photos	365 (43.4%)	14 (51.9%)	0.433		
≥40photos	476 (56.6%)	13 (48.1%)			
NF-NBI					
nonuse of NF-NBI	162 (19.3%)	22 (81.5%)	0.000	16.78 (5.50-51.26)	0.000
use of NF-NBI	679 (80.7%)	5 (18.5%)			
Atrophic gastritis					
Mild	538 (64.0%)	12 (44.4%)	0.039		
Moderate	185 (22.0%)	7 (25.9%)		1.17 (0.39-3.49)	0.781
Severe	118 (14.0%)	8 (29.6%)		2.49 (0.77-8.02)	0.127
Intestinal metaplasia					
Mild	683 (80.9%)	15 (55.6%)	0.005		
Moderate	74 (9.2%)	5 (18.5%)		3.41 (0.92-12.60)	0.066
Severe	84 (10.0%)	7 (25.9%)		3.84 (1.09-13.52)	0.036
Gastric mucosal inflammation					
Mild	557 (66.2%)	18 (66.7%)	1.000		
Severe	284 (33.8%)	9 (33.3%)			
Histologic type					
Differentiated	650 (77.3%)	24 (88.9%)	0.170		
Undifferentiated	191 (22.7%)	3 (11.1%)			

Notably, the sensitivity analysis using the 12-month definition yielded concordant results. Theassociations with nonuse of NF-NBI (OR: 15.09; 95% CI: 4.21–54.09) and severe intestinalmetaplasia (OR: 5.67; 95% CI: 1.33–24.12) remained strong and statistically significant. The association with the greater curvature location persisted, though with a wider confidence interval due to the reduced sample size (OR: 3.98; 95% CI: 0.97–16.33) (**Supplementary Table 1**).

#### 3.2.2 Risk factors for post-ESD diagnostic error

For post-ESD diagnostic errors identified within 24 months, significant risk factors included examination by trainees (OR: 3.53; 95% CI: 1.06–11.68), lesion size <15 mm (OR: 3.83; 95% CI: 1.10–13.36), nonuse of NF-NBI (OR: 17.44; 95% CI: 4.81–63.17), and severe atrophic gastritis (OR: 7.78; 95% CI: 1.91–31.20) (**Table 3**).

**Table 3:** Logistic regression analysis of risk factors associated with diagnosis error post-ESD.

Clinical parameters	EGCs without MEGCs (n=841)	Diagnosis error (n=22)	Chi-square	Multivariate analysis	
			P	Odds ratio (95% confidence interval)	P
Sex					
Male	637 (78.4%)	17 94.4%)	0.143		
Female	175 (21.6%)	1 (5.6%)			
Age					
<65years old	455 (44.0%)	5 (27.8%)	0.028	1.45 (0.35-5.93)	0.608
≥65years old	357 (56.0%)	13 (72.2%)			
Endoscopist					
trainee	326 (38.8%)	15 (68.1%)	0.007	3.53 (1.06-11.68)	0.039
expert	515 (61.2%)	7 (31.9%)			
Lesion size					
<1.5cm	373 (44.4%)	17 (77.3%)	0.002	3.83 (1.10-13.36)	0.035
≥1.5cm	468 (55.6%)	5 (22.7%)			
Lesion classification					
I	13 (1.5%)	0 (0.0%)	0.007		
IIa	93 (11.1%)	3 (13.6%)			
IIb	49 (5.8%)	5 (22.7%)			
IIc	521 (62.0%)	14 (63.7%)			
IIa+IIc	165 (19.6%)	0 (0.0%)			
Site 1					
Upper third	576 (68.5%)	17 (77.3%)	0.800		
Middle third	143 (17.0%)	3 (13.6%)			
Lower third	122 (14.5%)	2 (9.1%)			
Site 2					
Lesser curvature	408 (48.5%)	9 (41.0%)	0.688		
Greater curvature	66 (7.8%)	2 (9.0%)			
Anterior wall	98 (11.7%)	4 (18.2%)			
Posterior wall	269 (32.0%)	7 (31.8%)			
Mucosal visibility					
Low	10 (1.2%)	0 (0.0%)	0.329		
Medium	125 (14.9%)	6 (27.3%)			
High	706 (83.9%)	16 (72.7%)			
Quantity of image acquisition					
<40photos	365 (43.4%)	10 (45.5%)	1.000		
≥40photos	476 (56.6%)	12 (54.5%)			
NF-NBI					
nonuse of NF-NBI	162 (19.3%)	17 (77.3%)	0.000	17.44 (4.81-63.17)	0.000
use of NF-NBI	679 (80.7%)	5 (22.7%)			
Atrophic gastritis					
Mild	538 (64.0%)	5 (22.7%)	0.000		
Moderate	185 (22.0%)	5 (22.7%)		1.73 (0.37-7.97)	0.481
Severe	118 (14.0%)	12 (54.5%)		7.78 (1.91-31.20)	0.004
Intestinal metaplasia					
Mild	683 (80.9%)	13 (59.1%)	0.030		
Moderate	74 (9.2%)	4 (18.2%)		2.16 (0.50-9.44)	0.305
Severe	84 (10.0%)	5 (22.7%)		1.50 (0.30-7.55)	0.624
Gastric mucosal inflammation					
Mild	557 (66.2%)	9 (41.0%)	0.021		
Severe	284 (33.8%)	13 (59.0%)		2.82 (0.74-10.69)	0.127
Histologic type					
Differentiated	650 (77.3%)	20 (90.9%)	0.193		
Undifferentiated	191 (22.7%)	2 (9.1%)			

The sensitivity analysis (12-month window) confirmed the robustness of these findings, identifying lesion size <15 mm (OR: 15.07; 95% CI: 1.80–126.34), nonuse of NF-NBI (OR: 40.86; 95% CI: 4.90–341.40), and severe atrophic gastritis (OR: 11.42; 95% CI: 1.96–66.52) as key risk factors. The smaller sample size in this analysis led to wider confidence intervals, but the effect directions and significance of the core factors were strengthened or maintained (**Supplementary Table 2**).

## 4 Discussion

This study sought to systematically analyze missed diagnoses of EGC during the peri-ESD period by implementing an innovative “time-process bidirectional evaluation protocol” within a single-center cohort of the same patients. The key strength of this approach lies in its ability to go beyond merely reporting rates and, by examining both temporal and procedural dimensions, to precisely identify the underlying causes of diagnostic weaknesses at various stages. Our analysis revealed distinctive characteristics of missed lesions occurring in the peri-ESD period and predicted a series of adjustable risk factors. These findings offer valuable insights for developing targeted strategies to enhance quality, providing a solid foundation for future improvements.

When compared with existing literature, our peri-ESD assessment revealed an overall missed diagnosis rate of 10.1%, which differs from Yamamoto et al.’s broader population-based screening results, reflecting differences in study focus and inclusion criteria (14). In terms of conceptual alignment, our pre-ESD missed diagnosis rate of 5.6% was consistent with the trend reported by Yoshida et al. (7.6%), suggesting comparability across different assessment methods in identifying early-stage lesions (5).

Our findings suggest that during the peri-ESD phase, the patterns of missed diagnoses differed notably between the pre- and post-ESD stages. Pre-ESD omissions were primarily due to inadequate observation (51.9%), whereas post-ESD omissions more often resulted from diagnostic error (52.4%). This distinction has direct clinical implications: improving pre-ESD detection requires enhancing examination comprehensiveness, while reducing post-ESD errors demands better differentiation of subtle lesions.

Multivariate analysis identified nonuse of NF-NBI was associated with MEGCs. The interpretation of this finding is twofold. Firstly, within our study protocol, where NF-NBI was employed only after a suspicious lesion was detected on white-light endoscopy (WLE), failure to use NF-NBI most directly indicates that the lesion was missed during the initial white-light screening phase. This explanation is strongly supported by our data. Specifically, inadequate observation was the most common cause of pre-ESD missed lesions. These oversights frequently occurred at anatomically challenging locations such as the greater curvature, even among experienced endoscopists. These findings are consistent with established evidence regarding endoscopic blind spots (15) and highlight that deficiencies in the thoroughness of the baseline white-light examination are a major contributor to diagnostic oversight.

Secondly, nonuse of NF-NBI itself may directly lead to diagnostic errors in some instances. This is consistent with our analysis of factors associated with diagnostic error post-ESD, which identified diagnosis error was the most common cause of missed lesions after ESD. For example, an endoscopist might notice a subtle abnormality but, without utilizing NF-NBI for confirmation, misjudge it as benign. This decision-making bias would negate the diagnostic advantage of NF-NBI for EGC (16, 17).

Furthermore, specific mucosal backgrounds, such as severe intestinal metaplasia (IM) and atrophic gastritis, have been identified as independent risk factors. Patients with chronic IM exhibit elevated risks of dysplastic progression compared to non-metaplastic counterparts (18), with the condition demonstrating a higher odds ratio (OR) for neoplastic transformation than atrophic changes (19). IM demonstrates characteristic villiform patterns and reduced mucosal transparency under endoscopy (20). Following *H. pylori* eradication, these lesions typically evolve into erythematous geographic depressions (21), collectively masking early gastric carcinomas with ambiguous morphology in regions of metaplastic mucosal alteration (22, 23).

In the context of severe atrophic gastritis, mucosal pallor and altered vascular patterns make it easier for newly emerged, subtle neoplastic changes to be obscured. Persistent *H. pylori* infection increases the risk of metachronous gastric cancer or dysplasia after endoscopic resection (ER), though post-procedural eradication reduces this risk (24). Severe mucosal atrophy and follow-up durations exceeding five years have been identified as independent risk factors for metachronous cancer (25), with approximately 75% of such cancers being missed during initial diagnosis (26).

The *H. pylori* infection rate among Chinese individuals around 65 years of age is approximately 46–50% (27, 28). Among the 935 early gastric cancer cases analyzed in our study, 321 (34.3%) exhibited characteristic gastric mucosal inflammation changes—namely nodularity, diffuse erythema, and edema—indicative of current active *H. pylori* infection. This observed rate is relatively lower than the expected population-level prevalence, which may reflect both the effectiveness of recent eradication efforts and the specific characteristics of the clinical cohort. Real-world evidence indicates that about 57.6% of infected patients undergo eradication therapy, with bismuth-based quadruple therapy achieving an eradication rate of 76.7% (29), suggesting that some patients may have been successfully treated prior to endoscopic evaluation.

This study found that a lesion size <15 mm significantly increased the risk of post-ESD diagnostic errors, underscoring the difficulty in characterizing small lesions. This result corroborates the work of Zhang et al. (30), linking smaller size to higher miss rates. Similarly, insufficient operator experience was a key risk factor, emphasizing the role of expertise in lesion recognition. Converging evidence from Wang et al. (31) on standardized protocols and Kato et al. (32) on procedural volume solidifies the imperative to advance both training and standardization.

This study, conducted within a single-institution setting and using a consistent patient cohort, allowed for a systematic analysis while minimizing variations in endoscopic practices and diagnostic criteria. Building on this controlled foundation, we introduced a “temporal–procedural bidirectional assessment framework”. Although previous studies have provided foundational insights into the temporal and procedural aspects of missed gastric cancers (5, 15), our framework integrates these two dimensions to create a novel and clinically actionable tool. By mapping procedural failures onto specific temporal phases of the peri-ESD period, our approach not only identifies where and when errors occur but also illuminates how they propagate across the entire endoscopic cycle.

Based on our findings, we suggest several practical strategies to enhance detection: adopting NF-NBI as a routine tool in high-risk screening and post-ESD surveillance; refining standardized scanning protocols for high-risk anatomical sites and mucosal backgrounds; and strengthening specialized training in recognizing small and subtle lesions within complex mucosal environments. These approaches may contribute to improving the detection rate of early gastric cancer.

This study has several limitations. First, the retrospective design from a single tertiary center inherently introduces selection bias. Due to the limited sample size in certain subgroups, the multivariable analyses should be interpreted as exploratory. We plan to employ more robust statistical methods, such as Firth penalized regression, in future large-scale collaborations to build on this foundational work. Second, our analysis of missed diagnoses relied on static endoscopic images from historical databases, which lack dynamic mucosal assessment capabilities. Furthermore, the differentiation between “inadequate observation” and “diagnostic error” based solely on this retrospective review is inherently limited. Although our standard-based rigorous image review process was designed to ensure objectivity, it cannot accurately reconstruct the real-time judgment of the initial endoscopist. Third, the MEGC was defined as lesions detected within a 24-month peri-ESD window, based on the natural history of early gastric cancer where lesions remain early-stage for a median of 44 months (33). The robustness of this definition was confirmed by sensitivity analysis using a 12-month window, which yielded consistent results. Fourth, a major limitation is our reliance on an endoscopic surrogate for *H. pylori* status instead of gold-standard serology. The mucosal state influenced by *H. pylori* may affect lesion visibility, and our image-based design cannot exclude confounding by these unmeasured factors. Future studies with serological confirmation are needed to clarify this relationship. Fifth, the generalizability of our findings is limited to peri-ESD MEGCs that meet specific guideline criteria. These results may not apply to broader MEGC contexts, such as advanced-stage tumors (pT1b or deeper) or cases requiring additional surgery. Future multi-center studies are needed to validate these findings across diverse MEGC subtypes.

In conclusion, the missed diagnosis rate of early gastric cancer was 10.1%. Optimizing peri-ESD gastroscopic observation (via NF-NBI) and post-ESD diagnostic accuracy (through operator training) could significantly reduce peri-ESD MEGCs, particularly those reflecting characteristics of ESD-eligible lesions.

## Data Availability

The original contributions presented in the study are included in the article/**Supplementary Material**. Further inquiries can be directed to the corresponding author.

## Supplementary material

Supplementary Table 1 A sensitivity analysis of risk factors for inadequate observation in pre-ESD MEGCs using the 12-month definition. MEGC, missed early gastric cancer; ESD, endoscopic submucosal dissection.

Supplementary Table 2 A sensitivity analysis of risk factors for diagnosis error in post-ESD MEGCs using the 12-month definition. MEGC, missed early gastric cancer; ESD, endoscopic submucosal dissection.
