# Supplementary material for: Optimizing endoscopic detection of early gastric cancer: stratification and preventive strategies for Peri-ESD diagnostic oversights
Source: Front Oncol. 2026 Feb 10;16:1745307. doi: 10.3389/fonc.2026.1745307 (PMC12929169; doi:10.3389/fonc.2026.1745307)
Supplement: Supplementary Table 1 — A sensitivity analysis of risk factors for inadequate observation in pre-ESD MEGCs using the 12-month definition. MEGC, missed early gastric cancer; ESD, endoscopic submucosal dissection. [file Table1.docx]

**Supplementary table 1.** A sensitivity analysis of risk factors for inadequate observation in pre-ESD MEGCs using the 12-Month Definition.

|  | EGCs without MEGCs  (n=841) | Inadequate observation  (n=18) | chi-square | Multivariate analysis | |
| --- | --- | --- | --- | --- | --- |
|  |  |  | P | Odds ratio  (95% confidence interval) | P |
| Sex | | | | | |
| Male | 637 (78.4%) | 17 (94.4%) | 0.143 |  |  |
| Female | 175 (21.6%) | 1 (5.6%) |  |  |  |
| Age | | | | | |
| ＜65years old | 455 (44.0%) | 9 (50.0%) | 0.638 |  |  |
| ≥65years old | 357 (56.0%) | 9 (50.0%) |  |  |  |
| Endoscopist | | | | | |
| trainee | 326 (38.8%) | 10 (55.6%) | 0.221 |  |  |
| expert | 515 (61.2%) | 8 (44.4%) |  |  |  |
| Lesion size | | | | | |
| ＜1.5cm | 373 (44.4%) | 13 (72.2%) | 0.029 | 2.68（0.86-8.39） | 0.089 |
| ≥1.5cm | 468 (55.6%) | 5 (27.8%) |  |  |  |
| Lesion classification | | | | | |
| Ｉ | 13 (1.5%) | 0 (0.0%) | 0.004 |  |  |
| Ⅱa | 93 (11.1%) | 0 (0.0%) |  |  |  |
| Ⅱb | 49 (5.8%) | 6 (33.3%) |  |  |  |
| Ⅱc | 521 (62.0%) | 9 (50.0%) |  |  |  |
| Ⅱa+Ⅱc | 165 (19.6%) | 3 (16.7%) |  |  |  |
| Site 1 | | | | | |
| Upper third | 576 (68.5%) | 10 (55.6%) | 0.354 |  |  |
| Middle third | 143 (17.0%) | 5 (27.8%) |  |  |  |
| Lower third | 122 (14.5%) | 3 (16.7%) |  |  |  |
| Site 2 | | | | | |
| Lesser curvature | 408 (48.5%) | 7 (38.9%) | 0.007 |  |  |
| Greater curvature | 66 (7.8%) | 5 (27.8%) |  | 3.98 (0.97-16.33) | 0.050 |
| Anterior wall | 98 (11.7%) | 4 (22.2%) |  | 3.72 (0.89-1.63) | 0.073 |
| Posterior wall | 269 (32.0%) | 2 (11.1%) |  | 0.49 (0.94-2.56) | 0.399 |
| Mucosal visibility | | | | | |
| Low | 10 (1.2%) | 1 (5.6%) | 0.061 |  |  |
| Medium | 125 (14.9%) | 5 (27.8%) |  |  |  |
| High | 706 (83.9%) | 12 (66.7%) |  |  |  |
| Quantity of image acquisition | | | | | |
| ＜40photos | 365 (43.4%) | 9 (50.0%) | 0.635 |  |  |
| ≥40photos | 476 (56.6%) | 9 (50.0%) |  |  |  |
| NF-NBI | | | | | |
| nonuse of NF-NBI | 162 (19.3%) | 14 (77.8%) | 0.000 | 15.09(4.21-54.09) | 0.000 |
| use of NF-NBI | 679 (80.7%) | 4 (22.2%) |  |  |  |
| Atrophic gastritis | | | | | |
| Mild | 538 (64.0%) | 7 (38.9%) | 0.049 |  |  |
| Moderate | 185 (22.0%) | 6 (33.3%) |  | 1.56（0.45-5.36） | 0.483 |
| Severe | 118 (14.0%) | 5 (27.8%) |  | 1.84（0.41-8.17） | 0.423 |
| Intestinal metaplasia | | | | | |
| Mild | 683 (80.9%) | 9 (50.0%) | 0.003 |  |  |
| Moderate | 74 (9.2%) | 3 (16.7%) |  | 4.05（0.86-19.09） | 0.077 |
| Severe | 84 (10.0%) | 6 (33.3%) |  | 5.67（1.33-24.12） | 0.019 |
| Gastric mucosal inflammation | | | | | |
| Mild | 557 (66.2%) | 12 (66.7%) | 1.000 |  |  |
| Severe | 284 (33.8%) | 6 (33.3%) |  |  |  |
| Histologic type | | | | | |
| Differentiated | 650 (77.3%) | 17(94.4%) | 0.093 |  |  |
| Undifferentiated | 191 (22.7%) | 1 (5.6%) |  |  |  |
